# Quantitative assessment of pilot-endured workloads during helicopter flying emergencies: an analysis of physiological parameters during an autorotation

**DOI:** 10.1038/s41598-021-96773-y

**Published:** 2021-09-06

**Authors:** José Ricardo Silva Scarpari, Mauricio Watanabe Ribeiro, Camila Sardeto Deolindo, Maria Adelia Albano Aratanha, Donizeti de Andrade, Carlos Henrique Quartucci Forster, José Márcio Pereira Figueira, Fernando Lucas Soares Corrêa, Shirley Silva Lacerda, Birajara Soares Machado, Edson Amaro Júnior, João Ricardo Sato, Elisa Harumi Kozasa, Roberto Gil Annes da Silva

**Affiliations:** 1 Instituto Tecnológico de Aeronáutica, São José dos Campos, 12228-900 Brazil; 2 Instituto de Pesquisas e Ensaio em Voo (IPEV), São José dos Campos, 12228-900 Brazil; 3 Hospital Israelita Albert Einstein, Brain Institute, São Paulo, 01425-001 Brazil; 4 Universidade Federal do ABC, São Bernardo do Campo, Brazil

**Keywords:** Aerospace engineering, Cognitive neuroscience

## Abstract

The procedures to be performed after sudden engine failure of a single-engine helicopter impose high workload on pilots. The maneuver to regain aircraft control and safe landing is called autorotation. The safety limits to conduct this maneuver are based on the aircraft height versus speed diagram, which is also known as "Dead Man’s Curve”. Flight-test pilots often use subjective methods to assess the difficulty to conduct maneuvers in the vicinity of this curve. We carried out an extensive flight test campaign to verify the feasibility of establishing quantitative physiological parameters to better assess the workload endured by pilots undergoing those piloting conditions. Eleven pilots were fully instrumented with sensors and had their physiological reactions collected during autorotation maneuvers. Our analyses suggested that physiological measurements (heart rate and electrodermal activity) can be successfully recorded and useful to capture the most effort-demanding effects during the maneuvers. Additionally, the helicopter’s flight controls displacements were also recorded, as well as the pilots’ subjective responses evaluated by the Handling Qualities Rate scale. Our results revealed that the degree of cognitive workload was associated with the helicopter’s flight profile concerning the Height-Speed diagram and that the strain intensity showed a correlation with measurable physiological responses. Recording flight controls displacement and quantifying the pilot's subjective responses show themselves as natural effective candidates to evaluate the intensity of cognitive workload in such maneuvers.

## Introduction

Flight safety, comfort, efficiency, and performance are crucial to the development of new aeronautical projects as well as means of task automation, flight controls simplification, and piloting aids such as alarms and automatic flight control systems^1^. Therefore, the Human Factors must be considered in the conceptual and preliminary phases of the aircraft design, particularly concerning to the cabin and the cockpit. This consideration contributes to reduce the pilot’s workload^2^.

Within the aeronautical context, workload is defined as a measure of the attention and skill required from a pilot to accomplish a task, achieve the desired performance, and be able to carry out secondary tasks^3^. The pilot’s workload is the integrated cognitive and physical effort required to satisfy the demands of a specified flight task^4^, and this is subject to the increased difficulties of the operational situation, time pressure, atmospheric conditions, and usable cue environment (UCE)^5^.

The workload can be physical, in terms of control activity, or alternatively cognitive, in terms of concentration. In many cases, cognitive and physical demands increase concurrently. Cognitive workload emerges from the interaction between the operator with the environment and mental resources devoted during the execution of a specific task. Consequently, quantifying workload intensity poses some issue, such as the difficulty of standardizing the evaluation and the subjectivity of the results. Nevertheless, there are some traditionally used methods to infer cognitive workloads such as the pilots’ subjective ratings, psychophysiological responses, and performance^6–8^.

In helicopter piloting, autorotation is performed after an unexpected engine failure, and this represents one of the most complex maneuvers and imposes a high workload on the pilot. The pilot’s cognitive demands increase substantially to accomplish all the tasks required for safe landing especially in the case of single-engine aircraft^9^. The failure to carry out the needed procedures can be catastrophic because the engine no longer (or only partially) provides net power to the rotor systems. The main rotor’s speed abruptly drops with immediate problems such as (1) reduction of lift that affects the ability to maintain the flight leveled, (2) partial loss of control that demands a wider displacement of flight controls, increasing the pilot’s workload, (3) abrupt changes in bending moments compared to normal condition values with the risk of rupture of the root of the blades, and (4) the possibility to reach the lowest and unrecoverable Main Rotor RPM Speed^10^.

To assist the definition of the operational flight envelope, especially during an emergency, there is a diagram that employs aircraft height and speed combination to define unsafe flight conditions that is called Height-Speed Diagram or “Dead Man’s Curve”^11^. This diagram illustrates the difficulty of performing an autorotation maneuver. This is built by flight test pilots and engineers based on the evaluation of the aircraft’s ability to convert energy to keep the main rotor RPM speed, and the pilot’s workload required to complete the task. There is a lack of international standards for assessing the pilot’s workload in that condition. Thus, this diagram appears to be a possibility of having a methodology to measure the pilot’s workload objectively. Due to the many variables contributing to the workload, it is important to consider as many variables as possible^9^.

The Standard FAR-Part 27^12^, which applies to light helicopters with a maximum takeoff weight of up to 2741 kg (6000 lb), standardizes that the limiting of the Height-Speed envelope can be drawn from mathematical models that consider the transformation of translational kinetic and gravitational potential energy into main rotor system RPM (rotational kinetic energy). For this reason, the human factor attributes to perform this maneuver are traditionally disregarded^13^ as it just indicates that the maneuver shall not require exceptional piloting skills, which constitutes a current operational vulnerability.

Workload evaluation during complex maneuvers and tasks presents technical difficulties^14^ especially during emergencies such as the autorotation that are not only related to the pilot’s inherently short reaction time but also to the subjectivity of most commonly used workload assessment rating scales^15,16^ such as the Cooper-Harper^17^, the Bedford^18,19^, and the NASA-TLX^20^. Although they are the prevalent methods for workload measurements, intrinsic error-prone aspects to subjective ratings exist as they are influenced by cultural values^21^ and biases, especially in high-risk environments^16^.

Another workload assessment method aims at developing a technique based on the cognition and physiology interface. In this sense, there is a growing evidence pointing to psychophysiological signals to measure workload^22^. Such a proposal is less prone to subjectivity and the parameters are continuously sampled during the task execution. This approach is promising as the participant’s workload can be evaluated by the in-flight pilot’s anticipatory responses in addition to the after-task estimates^23^.

For this research, the results of an extensive Flight Test Campaign are taken and interpreted. A single-engine helicopter was subjected to several programmed engine failures and some unexpected, when only the instructor pilot knew when the engine failure would be simulated. During the tests, both the aircraft and the pilots were fully instrumented with sensors capable of capturing and recording the aircraft's flight conditions. In the aircraft we monitored speeds, accelerations, attitudes, flight controls displacements, engine power regime, and the pilot’s physiological reactions were also recorded.

During this Campaign we assessed the robustness of this method focused on the execution of autorotation maneuvers, while also verifying how the cognitive activity relates or results in physiological modulation. To this end, we collected and analyzed electrodermal activity (EDA), electrocardiogram activity (EKG), and respiration frequency as parameters to quantify the pilots’ workloads. In our study, the recordings of aircraft flight controls displacements during autorotation were simultaneous to the physiological sensors’ recordings.

This study presents the workload quantitative results obtained by exploiting the methodology that considers flight control displacements and physiological responses of pilots to complement with objective data to the traditional workload assessment, which is essentially subjective. This methodology functions as a potential guide for the development of new aeronautical projects, especially cockpit design, allowing a better understanding on how designers may optimize and reduce the workload of pilots, particularly in high psycho-motor and stress situations. To the best of our knowledge, this applied study is unique in the aviation area.

To assess the workload of simulated engine failures during a helicopter flight test campaign we used three different methods: the authors (1) applied an approach similar to that used in the Aeronautical Design Standard-33 (ADS-33)^24^, adapted the autorotation maneuvers as if they were a Mission Task Element (MTE) and employed the evaluation methodology typical of Flight Test Schools by using the Handling Qualities Rate (HQR) scale; they also (2) used the pilots’ physiological responses, and (3) evaluated the flight controls displacements during the maneuver (amplitude and frequency).

We further explored the areas of the Height-Speed Diagram and its association with the workload. The classic partitions involve the “outside” (safe) and “inside” (unsafe) areas of the curve. The former is related to flight conditions with a lower workload in which it is safe to perform an autorotation maneuver, and the latter comprises higher workload scenario that even skilled pilots would not be able to avoid a fatal crash. Considering the techniques required to perform an autorotation at four different areas (Take-off, Knee, Cruise, and High-hover) we hypothesize that another partition would provide further insights on workload assessments. These areas are detailed in the Method section. Then, we also tested whether pilots’ expertise would be a factor affecting the ability to perform the maneuver and the associated workload.

## Methods

**Figure 1:**
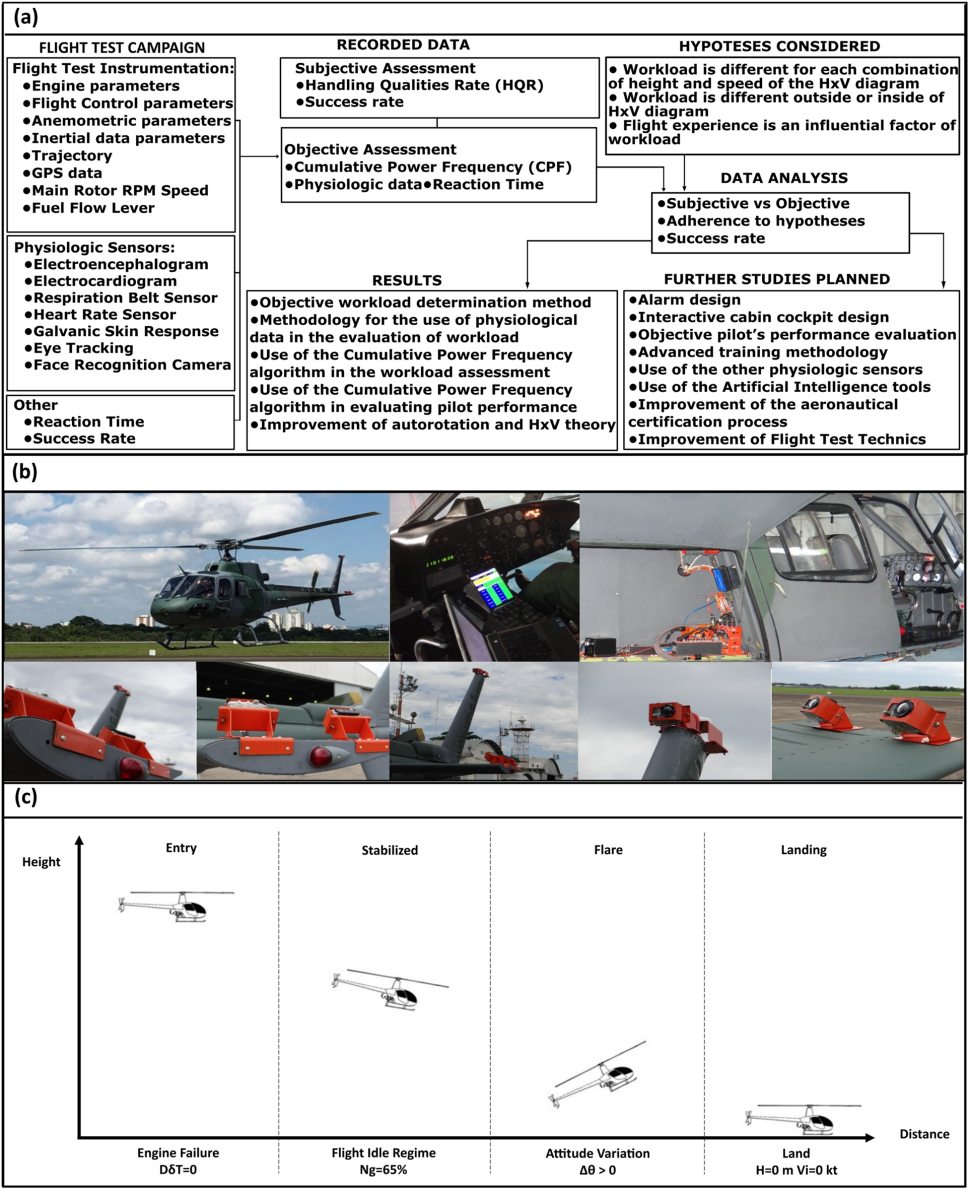
**(a)** Flowchart of the experiment and methodology proposed. **(b)** AS-350 helicopter and its Flight Test Instrumentation (FTI). **(c)** Stages of autorotation. Fuel lever control position (DδT). Turbine Gas generator Speed (Ng). Attitude variation (Δθ). Height (H). Airspeed (Vi).

The main objective of the Helicopter Flight Test Campaign was to obtain aircraft’s data, by means of Flight Test Instrumentation (FTI), and pilot’s data by taking advantage of dedicated physiological sensors. These data were analyzed and compared with the results of traditional methods to validate the hypotheses proposed in this research and to guide future studies, as shown in Fig. 1a.

### Participants

Eleven elite military pilots volunteered to participate in this campaign. All volunteers were helicopter flight instructors with more than 2000 h of flight experience. Although all pilots had considerable flight experience, based on the specific training required to perform autorotation maneuvers, we predicted that their subjective workload, physiological responses, and quantitative flight control recordings could be divided into three groups: (1) highly experienced test pilots i.e. autorotation instructors, with more than 3000 landings in full autorotation each; (2) test pilots, that had experience in flight testing, but with only a few hundred of full autorotations; and (3) operational pilots, military pilots who served in operational squadrons of the Brazilian Air Force and the Army with no experience in full autorotations. This division was adopted as a way of mitigating risks given that all flights had a highly experienced test pilot flying as a co-pilot.

The flight test campaign has been set to evaluate the pilot’s subjective responses at different partitions of the Height-Speed diagram as part of their work duties, the pilots’ physiological responses were recorded. The Ethical Committee of the Hospital Israelita Albert Einstein approved all procedures (CAAE 72744717.7.0000.0071). All volunteers who agreed to participate signed an informed consent form. This study was performed in accordance with all relevant guidelines and regulations.

### Flight campaign

A single-engine helicopter from the Brazilian Air Force model AS-350 with dedicated Flight Test Instrumentation (FTI) was used during all flights (Fig. 1b). The FTI had a 64 Hz rate of acquisition, and the flight data were recorded with the KAM-500 (RIPROG) equipment. The aircraft was instrumented with an Inertial Measurement Unit (IMU) which provides the measurement of angular velocity about the x-axis (*p*), y-axis (*q*), and z-axis (*r*), as well as pitch (*Θ*), roll (*Φ*), and yaw (*Ψ*) angles. There was also a system to measure pilot input commands, such as Collective (*Dδc*), Lateral (*Dδl*), and Longitudinal (*Dδm*) pitch control positions. Part of the equipment was also the Fuel lever control position (*DδT*) and quantity, the Engine gas generator speed (Ng), torque (Tq), and main rotor speed (Nr), complemented by GPS data, and internal/external cameras (Fig. 1b). A complete list of symbols is presented at the end of the manuscript (Table 1).

**Table 1:** List of symbols.

Symbol	Description	Unit
*Dδc*	Collective pitch control position	%
*Dδl*	Lateral pitch control position	%
*Dδm*	Longitudinal pitch control position	%
*Dδn*	Pedal pitch control position	%
*DδT*	Fuel level control position	%
*I*	Moment of inertia	kg m^2^
*KIAS*	Knots indicated airspeed	Kt
*Ng*	Engine gas generator speed	%
*N*_*r*_	Main rotor speed	RPM
*p*	Angular velocity about x-axis	°/s
*q*	Angular velocity about y-axis	°/s
*r*	Angular velocity about z-axis	°/s
*Tq*	Engine torque	%
*θ *(theta)	Pitch angle, shaft angle	°
*φ *(phi)	Roll angle	°
*ψ *(psi)	Yaw angle	°

Flight mechanics data responses were continuously recorded, and three main recording points were standardized: engine failure (when the engine stabilized in idle regime: Ng = 65%), autorotation’s entry (when the pilot started the movement to lower the position of the collective control), and at the start of the Flare (when the variation of the aircraft’s longitudinal attitude started to become positive, already close to the ground: ∆*θ*  >  0). During the flight, the parameters were managed by the pilots with entry into autorotation as soon as the Ng reached 65%. The Flare was started at 65ft in height and the preparation for landing between 20 and 25ft, according to the flight manual of aircraft. The analysis of the data recorded by the FTI was analyzed with parameters obtained automatically by Matlab scripts. These points fulfill the three stages of autorotation, i.e., (1) entry into autorotation, (2) stabilized autorotation, and (3) flare and landing (Fig. 1c).

**Figure 2:**
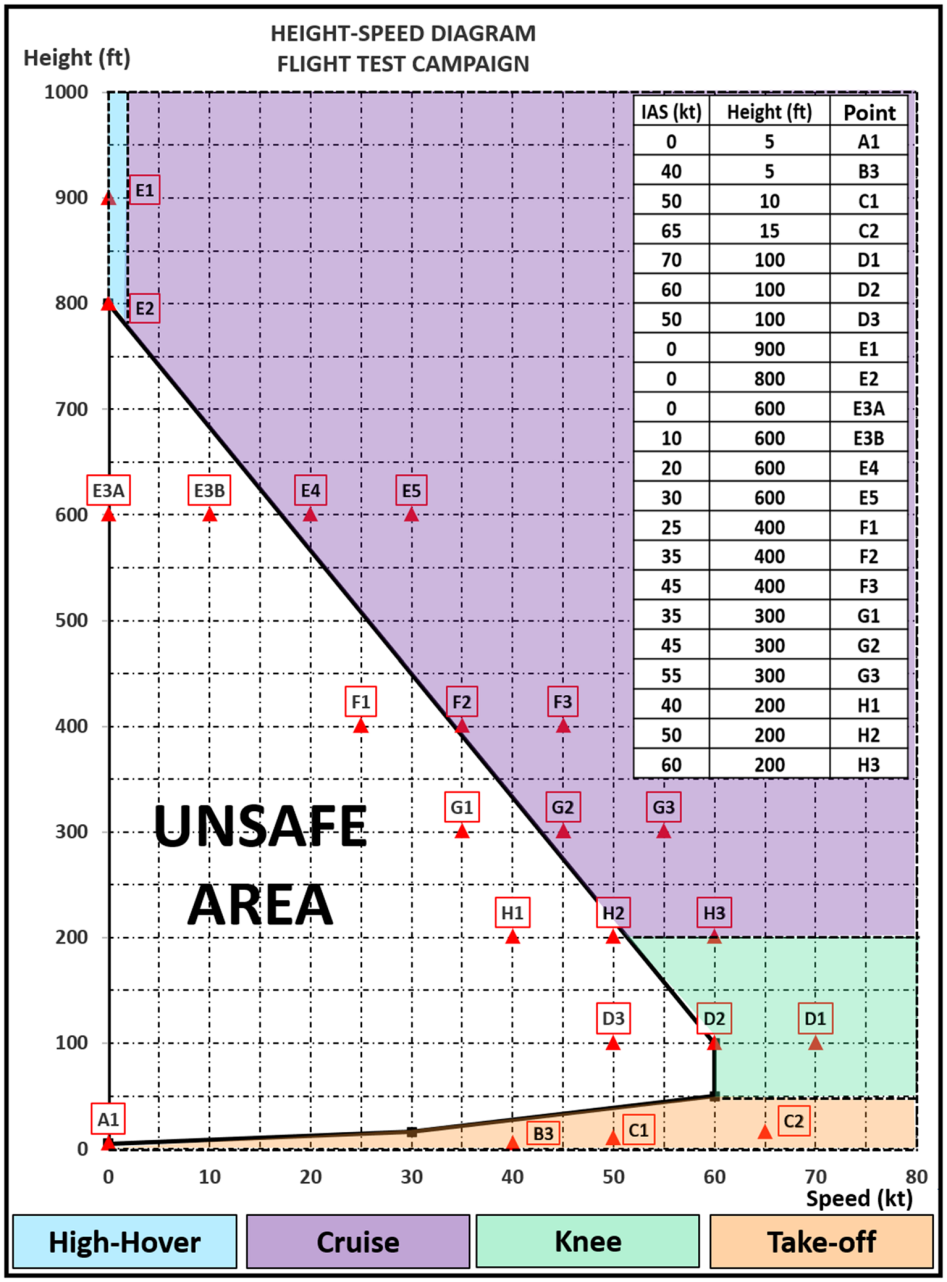
Height-Speed Diagram and the four partitions proposed, i.e., High-hover, Cruise, Knee, and Take-off. The table in the upper right corner shows the height and speed combinations tested during the test campaign as well as their different encodings (Letter-Number).

As mentioned before, one of the most complex maneuvers for a helicopter pilot to perform, an autorotation, is expected to be done after an unexpected failure, especially in a single-engine aircraft. This maneuver imposes a high workload on the pilot who must perform all the tasks necessary for safe landing^9^. The Height-Speed diagram (Fig. 2) helps in the assessment of the associated workload. This curve defines the unsafe flight conditions regarding solely aircraft speed, height and the consequent difficulty of performing an autorotation^11^ . These conditions can provide the basis for selecting different levels of difficulty to perform the maneuver. Therefore, whereas “outside” the curve it may be generally safe to perform the autorotation, however, when “inside”, not even skilled pilots would be able to avoid a fatal crash.

Each pilot conducted between 12 and 27 complete autorotations at different height and speed combinations, which corresponded to different complexity levels and expected workloads. The corresponding autorotations are depicted with red triangles in Fig. 2. The fuel lever was reduced to the flight idle position (Ng = 65%) to simulate engine failure. At least one maneuver was performed unexpectedly.

Each pilot performed two flights, except pilot #6, who completed all the maneuvers in one flight. We define and assessed two different classifications between the performed maneuvers (Fig. 2).

Classification (a) conformed the classic distribution including points Outside [O] (C1, C2, D3, E1, E5, F3, and G3), points in the limit Line [L] (D2, E2, E4, F2, G2, and H2) and points inside the Height-Speed diagram [I] (D1, E3A, E3B, F1, G1, and H1).

Classification (b) conformed the partition into four areas considering types and maneuvering techniques required for each of these partitions, i.e., the Take-off (B3 and C1), the Knee (D1, D2, D3, H1, H2, and H3), the Cruise (G1, G2, G3, F1, F2, F3, E3B, E4, and E5), and the High-hover (E1, E2, and E3A). Point A1, although tested, was not part of the analysis as the aircraft touches the ground in less than a second, and had no time to fully use the flight controls or to measure physiological changes.

### Performance quantification

The first task of this research involves the determination of the quantitative criteria to measure the workload during autorotation maneuvers while maintaining the same performance standard for all pilots. Among all the existing models, we have decided to use as a basis for the study the same methodology of the Mission Task Elements (MTE) adopted in Aeronautical Design Standard-33 (ADS-33)^24^.

This standard defines criteria and specifications for handling qualities for a range of helicopters from scout and attack to utility and cargo^25^. Among all the quantitative and qualitative methods of ADS-33, there are the definitions of the tasks that must be evaluated with a performance standard that result may reach the desired or adequate level. The ADS-33 does not mention emergency maneuvers such as autorotation. However, as the method was already known and applied by the test pilots involved in our study, we decided to adapt the method by creating the MTE based on the foundations of the development with the original ADS-33, as described in the document Test Guide for ADS-33 PRF^26^ and the Background Information and User’s Guide (BIUG) for handling Qualities Requirements for Military Rotorcraft^27^, that adapted the autorotation tasks for a type of MTE.

The creation process of this autorotation’s MTE was studied, developed, and tested in more than 3000 simulated autorotations carried out at the Brazilian Air Force Flight Test School (IPEV). The results were presented and discussed at the 2014 and 2019 European Rotorcraft Forum^28,29^ and the 2015 American Helicopter Society Annual Forum^30^. The conditions imposed in this development considered that the aircraft should not exceed any structural limits during the maneuver to remain within the flight envelope limits approved by the aircraft manufacturer and allow the complete maneuver to be carried out safely.

We consider all influential factors of the helicopter's autorotation performance to be those resulting from the aerodynamics and efficiency of the kinetic and gravitational potential energy conversion into main rotor RPM speed (Nr)^28,31^. The most important factors studied were those related to the vertical and horizontal speed at the time of landing. Vertical speed is the most important and can present greater potential risks of a serious accident with the helicopter by generating decelerations above 20G^32,33^. In the study we decided that the vertical speed would not be considered as a performance but a safety criterion, with a maximum vertical speed of 2 m/s to be adopted.

Pilots were assessed regarding the workload required to perform each complete emergency landing, according to the HQR scale^17^ and their performance (desirable or adequate) according to our proposed MTE guideline^29^ (Table 2). The maneuver was considered successful if the pilot was able to accomplish it within parameters classified as “desirable” or “adequate”, without the mechanical or verbal intervention of the instructor.

**Table 2:** Mission task element (MTE) for autorotation, adapted from ADS-33. Used for workload assessment.

Engine failures occurring with airspeed (*V*_*i*_) ≥ 55 kt	Engine failures occurring with airspeed (*V*_*i*_) < 55 kt
Carry out the complete landing in autorotation Reach the recommended speed of autorotation (65 kt) Achieve the recommended RPM for autorotation Vertical speed at the time of landing ≤ 2 m/s Perform the complete maneuver with HQR ≤ 4.5	Carry out the complete landing in autorotation Maintain RPM above the minimum recommended Vertical speed at the time of landing ≤ 2 m/s Perform the complete maneuver with HQR ≤ 4.5
Desirable Performance: landing (touch the ground) at speeds less than 30 kt	
Adequate Performance: landing (touch the ground) at speeds less than 40 kt	

To better define the HQR of each task, the pilots performed the maneuvers with the “pilot-in-the-loop” method, and they were asked to perform a maneuver without exceptional piloting skills, i.e., to perform the maneuver in the same operational conditions that are typical of a helicopter instruction flight. The flight test engineers, who coordinated the execution of the flights, were instructed to conduct reviews of the HQR score of each maneuver, encouraging the pilots to comment on all their impressions to carry out the task. Therefore, the pilots further refined the HQR scores aided by a list of influential features, such as the field of vision, vibration, turbulence, and the most used flight controls displacements and eventual oscillations induced by the pilot (PIO), as recommended by ADS-33 Test Guide^34^.

The pilots were instructed to try the desirable performance. In the event of impossibility to conduct the maneuver, this was repeated to achieve at least adequate performance. All participants underwent previous training in flight simulators and real aircraft.

Although not usual, if the pilot managed to achieve the desired performance, even with a high workload, the grade HQR = 4.5 was used. This methodology is often used in flight tests in Brazil and it is provided in the EFEV manuals^3^, as well as in the BIUG^27^ and ADS-33 test Guide^33^. These Standard recommend two further criteria, the first requires that tasks to be performed by at least three test pilots (five would be preferable) with experience in the task, and the second specifies that the HQR standard deviation needs to be reduced with prior training of pilots in the tasks. We adopted a deviation limit of 1-unit of HQR among pilot’s evaluations.

We assessed the Success Rate (SR) of each group considering: (a) all test points; (b) only reasonable points (limit and outside). In addition, the pilots’ reaction time at each maneuver were recorded. The reaction was defined as the interval between the simulated engine failure and the onset of the pilot’s reaction on the flight controls, especially at lowering the collective control.

### Flight control quantification: cumulative power frequency

An important criterion to be evaluated in the development of an aircraft is the flight qualities, defined by Padfield as: “ease and precision with which a pilot can perform the tasks required in support of an aircraft role”^5^. One of the possible ways of evaluating these flight qualities is related to the frequency and amplitude in which the pilot uses the aircraft's flight commands. In this sense, during higher workload scenarios such as autorotation, these inputs can present larger displacements, which translates into higher frequencies and/or larger amplitudes applied to the flight controls.

A set of parameters broadly evaluated during a flight is the pilot’s action timing at manipulating or activating the flight controls, as well as the amplitude and frequency of each input. When the pilots use larger and high frequency flight control inputs, they must actively consider when, how and where these inputs must be made to accomplish the required task with satisfactory levels of performance and safety. In this sense, during higher workload scenarios, the input commands present larger displacements, which translates into higher frequencies and larger amplitudes applied to the flight controls.

To quantify the performance at flight controls, we employed the Cumulative Power Frequency (CPF)^35^ algorithm, making use of control displacements associated with the four helicopter’s flight-control axes: collective, cyclic (longitudinal and lateral), and pedals. To determine the performance, the CPF of each controls axis was calculated separately using Eq. (1) and the sum of its results for the four axes that represented the CPF which consolidates the information from all flight controls in a single parameter and does not privilege the contribution of any of the helicopter’s controls. This is consistent with the helicopter piloting activity, which does not present a preferential control axis nor control axis coupling.

1 $${\omega }_{cum}=\frac{{\omega }_{cutoff}\cdot \frac{1}{2\pi }{\int }_{0}^{\infty }{G}_{\delta \delta }\cdot d\omega }{10}$$

where, ${\omega }_{cum}$ is the cumulative power frequency of one controls axis in the time window. ${\omega }_{cutoff}$ is the cut-off frequency in the time window; and ${G}_{\delta \delta }$ is the cumulative energy over the frequency range in the time window.

This measure was derived from a so-called Power Frequency metric^36^ that introduced the effect of the cumulative energy over the whole range of frequencies in a moving 3-s time window to reduce the pilot's influence on the values of power frequency^35^.

This tool was later adapted for a workload assessment process for shipboard approach and landing tasks and used only results from flights performed on simulators. This previous study was successful in predicting the Ship-Helicopter Operating Limitations (SHOL) based on pilot model predictions, and defined boundaries between acceptable and unacceptable workloads, according to Cumulative Power Frequency results^35^.

Basically, CPF correlates frequency, represented by the cut-off frequency, and intensity of pilot input in one specific axis, which is represented by the level of cumulative energy on the entire frequency range, time windows, and higher values relate to an increased number of commands applied to the aircraft controls^25^.

Initially, we computed the CPF of each autorotation performed by each pilot. This measurement was computed in the interval ranging from 5 s preceding the engine failure until 15 s afterward. Subsequently, these results were combined into groups, considering the average of the points used in the analyses.

### Physiological data

Several physiological sensors were attached to the pilots’ bodies during the Flight Campaign and data were recorded from electroencephalograms (EEG) (LiveAmp 32 active channel, Brain Products), electrocardiograms (EKG) (BIP2AUX adapter Gain 100, Brain Products) as well as the skin electrodermal activity (EDA) (GSR module, Brain Products), the respiration rates (Respiration Belt, Brain Products), and eye movements (tracker Tobii Pro Glasses 2). All sensors but eye movements were recorded at 500 Hz by means of an EEG amplifier and connection box (Live Amp Sensor & Trigger Extension, Brain Products) and they were synchronized with the aircraft Flight Test Instrumentation by means of a push-button, triggered at each maneuver. Here we report on the analyzed data from the EDA, and heartbeat and breathing frequency of the pilots. The EEG data is presented elsewhere^37^.

**Figure 3:**
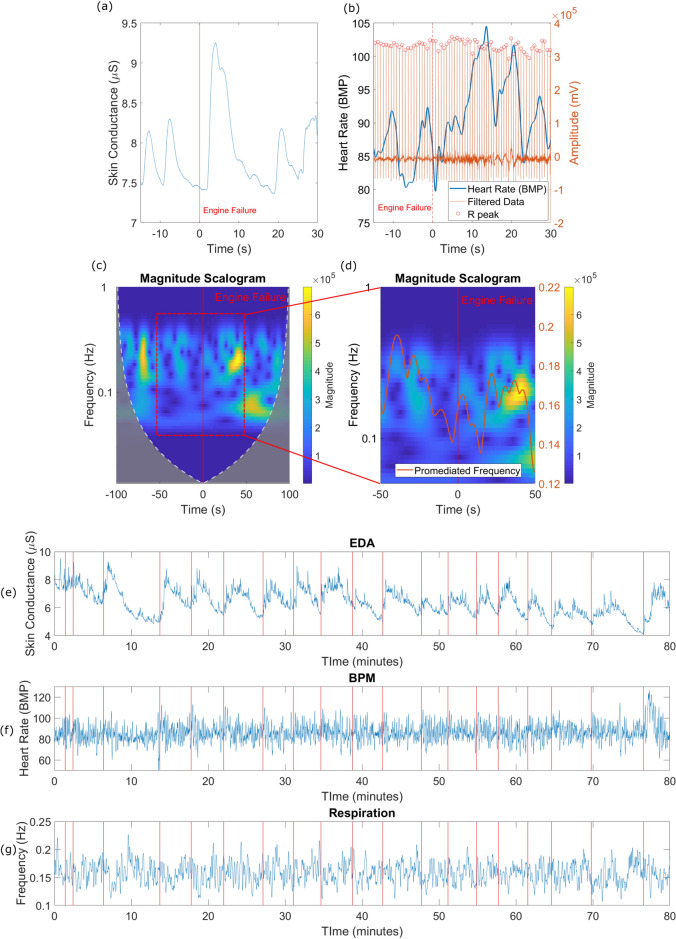
**(a)** Example of skin conductance response after engine failure. **(b)** Peak detection and heart rate computation. **(c)** Example of respiration scalogram around engine failure. **(d)** Zoom on the spectrogram at the frequency of interest and the promediated frequency calculated. The blue means the lowest magnitude value and red the highest. Example of the 3 physiological signals for a full flight. **(e)** EDA, **(f)** heart rate frequency, and **(g)** promediated respiratory frequency. The red vertical lines are the occurrence of engine failures.

Initially, for visual purposes, the raw EDA signals were segmented around each engine failure (− 60 s to 60 s) and normalized by the mean values computed in the 5 s before to the engine failure. We presented the mean waveforms in the result section. The related statistics were calculated using a linear mixed-effect model, as described in the next section. For statistical analysis, the amplitude of the first peaks after the event was selected for evaluation (Fig. 3a). We related these peaks to the engine failure only if the arousal response was initiated within a 0.5–5 s’ interval, as recommended in previously published studies^38^.

As for the EKG, the signals were initially band-pass filtered (1–100 Hz—zero phase, Fig. 3b), and we identified semi-automatically the R-peaks. In sequence, we computed the cardiac rate. Given the dissimilar basal frequencies at the onset of each maneuver, we normalized the heart rate by the mean value of the 5 s window prior to the engine failure. After that, we consistently observed modulation of the heart rate, consisting of two peaks as mean results for all maneuvers within 40 s after the engine failure. Therefore, to quantify this variation we computed, for each maneuver, the area under the curve (AUC) of the percent change and used the value for the statistics calculation by the linear mixed model.

Finally, the respiratory recordings were band-pass filtered (0.05–0.50 Hz, zero phase) and we attempted to characterize its rhythmical frequency. We computed the signal spectral activity using the wavelets transform with the use of a Morlet kernel. Due to the temporal uncertainty around the limits of the signal specially for the low frequencies (Fig. 3c), we adopted a − 100 s to 100 s window around each engine failure to calculate the frequency in the 0.05–0.50 Hz bandwidth from − 50 s to 50 s around the engine failure. For each instant, we normalized the mean power of each frequency band by the overall spectral power at that time, therefore, resulting in a premeditated frequency (Fig. 3)—(a) Example of skin conductance response after engine failure. (b) Peak detection and heart rate computation. (c) Example of respiration scalogram around engine failure. (d) Zoom on the spectrogram at the frequency of interest and the promediated frequency calculated. The blue means the lowest magnitude value and red the highest. Example of the three physiological signals for a full flight. (e) EDA, (f) Heart Rate Frequency, and (g) Promediated Respiratory Frequency. The red vertical lines are the occurrence of engine failures.

$${f}_{promediated}(t) = \frac{{\sum }_{i=0.05}^{0.50}{Spectral\,Power}_{i,t}\cdot {freq}_{i}}{{\sum }_{i=0.05}^{0.50}{Spectral\,Power}_{i,t}}$$

Equation (2) and Fig. 3d. Our goal by using this approach was to understand the instantaneous dynamics of the respiratory frequency in response to the engine failure. The dynamics of the recorded signals over a complete flight can be seen in Fig. 3e–g.

2 $${f}_{promediated}(t) = \frac{{\sum }_{i=0.05}^{0.50}{Spectral\,Power}_{i,t}\cdot {freq}_{i}}{{\sum }_{i=0.05}^{0.50}{Spectral\,Power}_{i,t}}$$

### Statistical analysis

We tested the modulation of each feature of interest (Cumulative Power Frequency, Electrodermal Activity, and Heart Rate) on the hypothesized partitions of the Height-Speed diagram with linear-mixed effect (LME) models. Each model started with intercept and random effects only and then, the group (Group1, Group2 or Group3), the classical (Inside, Line or Outside), or proposed (Cruise, High-hover, Take-off, or Knee) partition of the diagram was added in a second model. This new parameter survived in the model if it decreased the Akaike’s Information Criterion (AIC), and it was statistically significant after an ANOVA test^39^. The residual distribution of all models was assessed for Gaussian distribution and in case that the null hypothesis was rejected, a logarithmic operator was applied to the model expression. Models were fitted to the data using the maximum likelihood for model comparisons, and, for the results, we fitted the model with Restricted Maximum likelihood. Additionally, the difference between the levels of each significant factor was tested for significance with a false discovery rate correction. All statistical analyses were performed with the R 2020 software^40^ and packages^41–44^.

## Results

### Performance assessment

The results of the Handling Qualities assessment of all points evaluated by the pilots during the Flight Test Campaign, as well as the averages, and standard deviations of the groups are shown in Table 3.

**Table 3:** Results of the handling qualities assessment. Group 1—highly experienced test pilots; Group 2—test pilots; Group 3—operational pilots. Numbers in italics represents a failure to meet the BIUG requirement^27^.

Pilot’s experience	Pilot	Test points	Take-off			Knee						Cruise									High-hover		
			B3	C1	C2	D1	D2	D3	H1	H2	H3	G1	G2	G3	F1	F2	F3	E3B	E4	E5	E3A	E2	E1
			O			I	L	O	I	L	O	I	L	O	I	L	O	I	L	O	I	L	O
Test pilots Autorotation instructor (Group 1)	#1	HQR	6	5	3	8	5	4	9	7	5	4.5	4	4	5	4.5	4	4.5	8	3	3	4	8
	#2		5	4.5	4.5	7	4.5	4	7	7	4	6	4.5	5	7	–	4	4.5	6	–	4	4	6
	#6		4	4	5	6	6	5	9	5	5	4	4	5	4	4	3	5	7	4	5	5	7
Test pilots (Group 2)	#4		5	5	5	6	4	5	–	7	6	7	6	5	6	5	–	5	7	4	5	5	7
	#5		–	–	–	–	–	–	–	–	7	7	6	4	6	7	6	6	–	5			–
	#3		6	6	5	8	5	5	9	6	5	7	4.5	–	5	4.5	4.5	4	9	3	3	4	9
	#7		4.5	4	5	–	–	–	9	5	5	8	6	5	–	–	5	7	8	5	4	4.5	8
Operational pilots (Group 3)	#8		6	4.5	6	8	7	6	10	6	6	–	–	10	–	10	4.5	8	8	5	6	6	8
	#9		6	4	4	9	8	7	–	–	–	10	6	5	7	7	5	–	9	4	6	8	9
	#10		5	6	6	7	5	5	8	6	4	8	6	4	–	8	6	6	9	4	5	5	9
	#11		6	5	3	8	5	4	9	7	5	4.5	4	4	5	4.5	4	4.5	8	3	3	4	8
Average (all)			5.5	4.8	5.0	7.5	5.0	5.0	9.0	6.0	5.0	7.0	5.5	5.0	6.0	7.0	4.8	5.5	4.5	4.0	8.0	5.0	4.5
Average (test pilots)			5.0	4.8	5.0	7.0	5.0	5.0	9.0	6.5	5.0	7.0	4.5	5.0	5.5	4.5	4.3	5.0	4.5	4.0	7.5	4.0	4.0
Standard deviation (all)			*1.6*	0.7	0.8	1.0	*1.6*	*1.5*	0.9	0.8	0.9	*1.9*	0.8	*1.7*	1.0	*1.9*	1.0	*1.6*	0.4	0.7	*1.1*	*1.2*	1.0
Standard deviation (test pilots)			0.7	0.7	0.7	0.9	0.7	0.5	0.8	0.9	0.9	*1.4*	0.9	0.5	1.0	1.0	0.9	1.0	0.4	0.8	1.0	0.5	0.8

The highly experienced test pilots with more proficiency at dealing with autorotation had the best performances at the flight test campaign, achieving the highest success rates and effectively performing all autorotation maneuvers in the line limit (L) and outside (O) points. The overall average of the reaction over time was 1.18 ± 0.31 s (Table 3).

Analyses of workload data showed that the standard deviation (SD) was greater than one HQR unit when considering all the ratings from all pilots, representing a failure to meet the aeronautical BIUG requirement^27^ requirement in 38% of cases (Table 3). However, when the analyses were limited to the group of test pilots (Group 1 and 2) this criterion was met in 96% of the tested points. In fact, the SD was less than or equal to one HQR unit for 20 of the 21 test points, except at point G1 with an SD of 1.2. Although outside the standard limit, this point is inside of the Height-Speed diagram and this result is consistent with the pilots’ inability to perform the maneuver adequately. Therefore, the subjective workload evaluation only considered the response of the test pilots (#1 to #7). The wide dispersion of the evaluation carried out using the Bedford scale proved to be impractical.

Considering the four partitions of the Height-Speed diagram, the HQR averages (HQRav) followed the following pattern: (Cruise = 6.2) < (High-hover = 5.2) < (Cruise = 5.0) < (Take-off = 4.9). The averages of the points made in the traditional partitions (inside, line and outside) had the following pattern: (O = 4.7) < (L = 4.8) < (I = 6.8).This progressive pattern was also confirmed in all proposed Height-Speed diagram areas: Cruise (O = 4.4) < (L = 4.8) < (I = 5.6); High-hover: (O = 4.0) < (L = 4.4) < (I = 7.3) and Knee: (O = 4.9) < (L = 5.5) < (I = 7.8).

**Figure 4:**
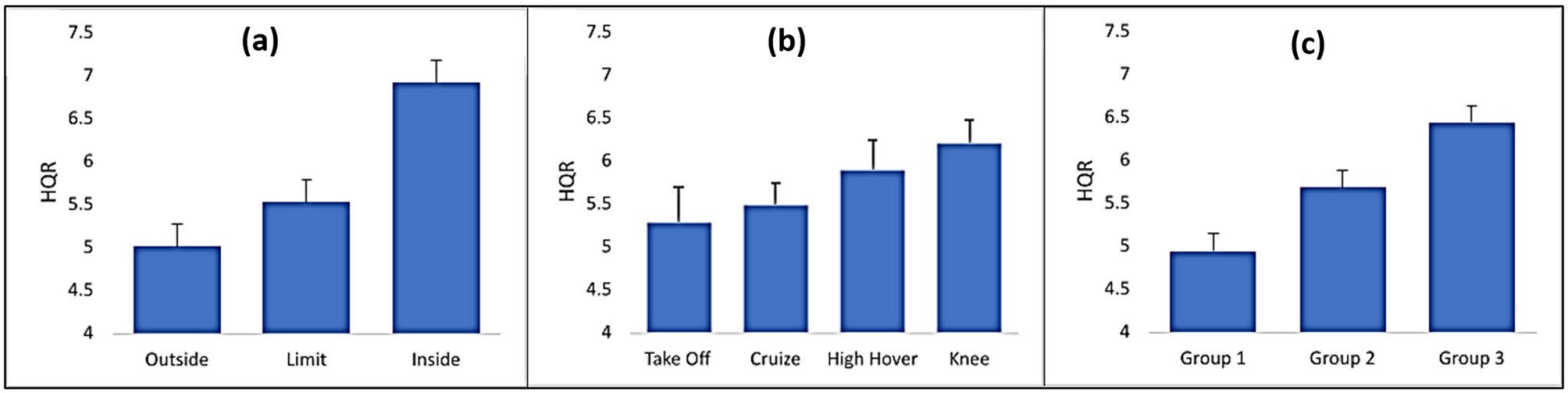
HQR values (mean and SEM) obtained by helicopter pilots submitted to an autorotation test at Height-Speed diagram. **(a)** All pilots grouped according to classical segmentation, **(b)** all pilots grouped according to the proposed segmentation, and **(c)** global.

In addition to the aeronautical requirement, we analyzed the HQR with linear mixed models. The main estimates are presented in Fig. 4.

The classical segmentation of the Height-Speed diagram presented the expected fit with the HQR values, with subjective workload values being higher at inside [(Inside > Line: EST = 1.58; p < 0.0001), (Inside > Outside: EST = 2.083; p < 0.0001)], followed by the workload at the line (Line > Outside: EST = 0.499; p = 0.0247). The proposed separation in areas were only different between Cruise and Knee (Knee > Cruise: EST = 0.786; p = 0.0169) and a trend between Knee and Take-off (Knee > Take-off: EST = 0.934; p = 0.0555). For the experience differences, the group with less experience reported more subjective workload [(Group 3 > Group 2: EST = 0.969; p = 0.0089) and (Group 3 > Group 1: EST = 1.5418; p = 0.0024)], and the groups of test pilots presented a trend between them (Group 2 > Group 1: EST = 0.548; p = 0.0872).

### Cumulative power frequency

**Figure 5:**
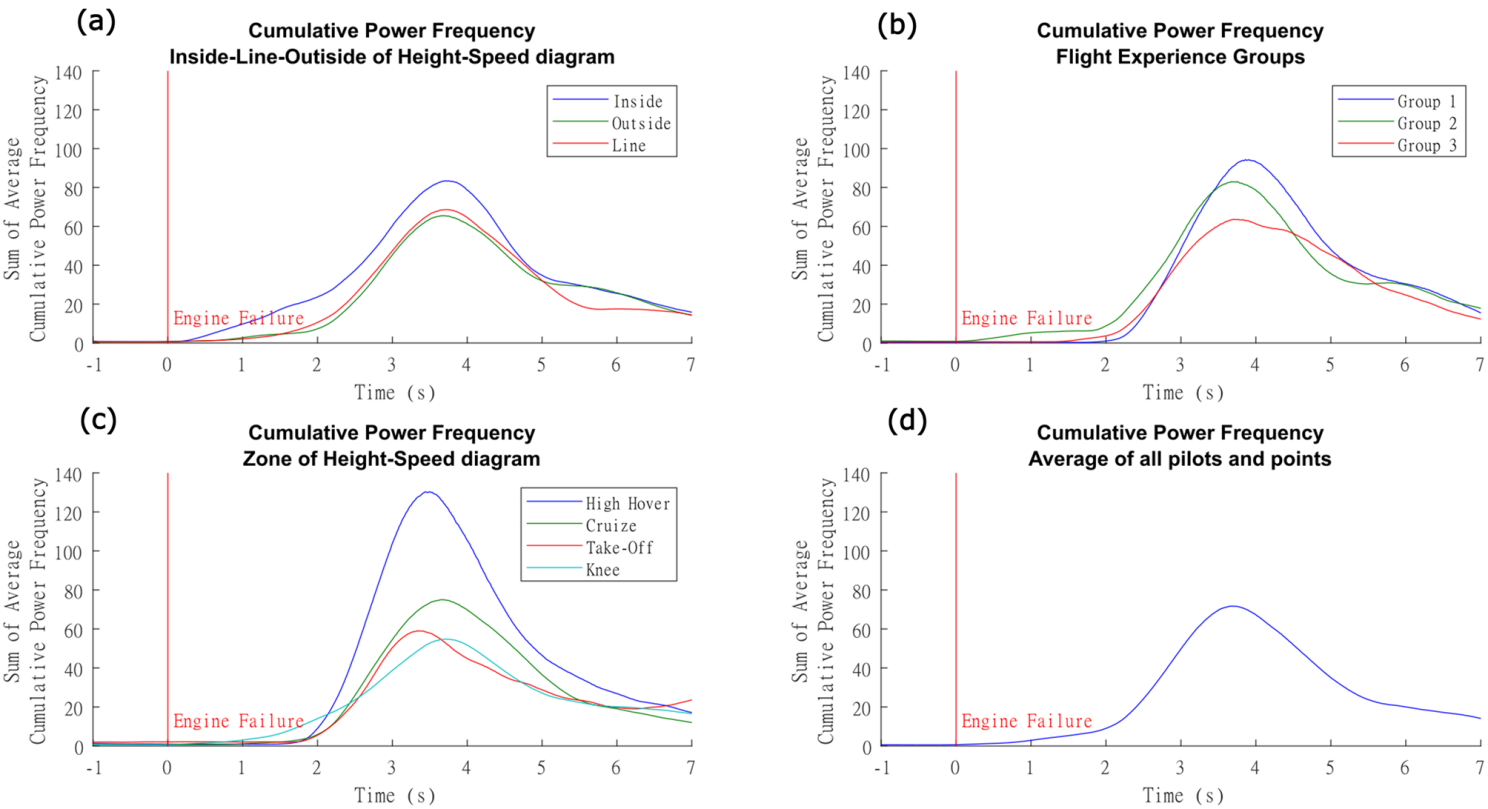
Cumulative Power Frequency of helicopter pilots carrying out in flight autorotation at height-speed diagram. **(a)** Pilots’ mean CPF at each phase of the classical partition of the height-speed diagram (inside, outside or at the line limit). **(b)** Mean cumulative CPF of different groups of pilots according to their previous experience: Group 1 (test pilots with extensive autorotation experience), Group 2 (test pilots with lesser autorotation experience) and Group 3 (operational pilots with lesser autorotation experience). **(c)** Mean CPF of all pilots at the four proposed zones. Notice a difference between High-hover and Cruise and a similarity between Take-off and Knee zone. **(d)** Sum of the global CPFs of all flights, indicating peak CPF responses at the time of engine failure. We emphasize that these averaged figures are used only to facilitate an overview of the data. For statistics purposes, the effects of repetitions were incorporated in a linear mixed effect model.

The first objective was to assess whether the CPF can be used as a parameter of workload right after the engine failure. We showed the average waveform of CPF after the engine failure in Fig. 5d. These graphics point that, on average, Inside (I) maneuvers have higher CPF than Outside (O) and Line (L). The Group 3 has lower CPF than Groups 1 and 2, and than the partition proposed has very distinct CPF patterns. However, we emphasize that the statistical analyzes utilized the peak value after the engine failure.

With the classical partition of the Height-Speed diagram (Inside, Line, or Outside), the maneuvers performed inside the curve had higher values than those performed outside (Fig. 5a). The CPF inside the curve was higher than outside (EST = 21.17 p = 0.0158) and outside was lower than at line (EST = 15.7 p = 0.0487). Thus, we showed that the CPF was able to identify objectively the workload points and distinguish the combinations of height and speed inside (I) and outside (O) of the Height-Speed diagram.

For the partition of the Height-Speed diagram in four areas (Fig. 2), the highest values were observed for High-hover followed by gradual reduction at Cruise, Take-off, and Knee, Fig. 5(c). The differences found are statistically significant for most of the partitions [High-hover > Cruise (EST = 63.9, p < 0.0001); High-hover > Knee (EST = 89.2, p < 0.0001); High-hover > Take-off (EST = 100.0, p < 0.0001); Cruise > Take-off (EST = 36.1, p < 0.001), Cruise > Knee (EST = 25.3, p =  < 0.0001)]. The exception was for the Knee zone which did not differ from Take-off (Knee-Take off = 10.8, p = 0.1644).

Regarding experience, there were no significant CPF peak differences among groups (Fig. 5b). The individual values for all models can be seen in the supplementary material files.

### Physiological measurements

Due to technical reasons, we lost all data from one flight of pilot #2 and the EKG from one flight from pilot #7. Therefore, we analyzed data from 190 maneuvers performed in 20 flights for EDA and respiratory frequency, and from 184 maneuvers in 19 flights for heart rate and EKG.

**Figure 6:**
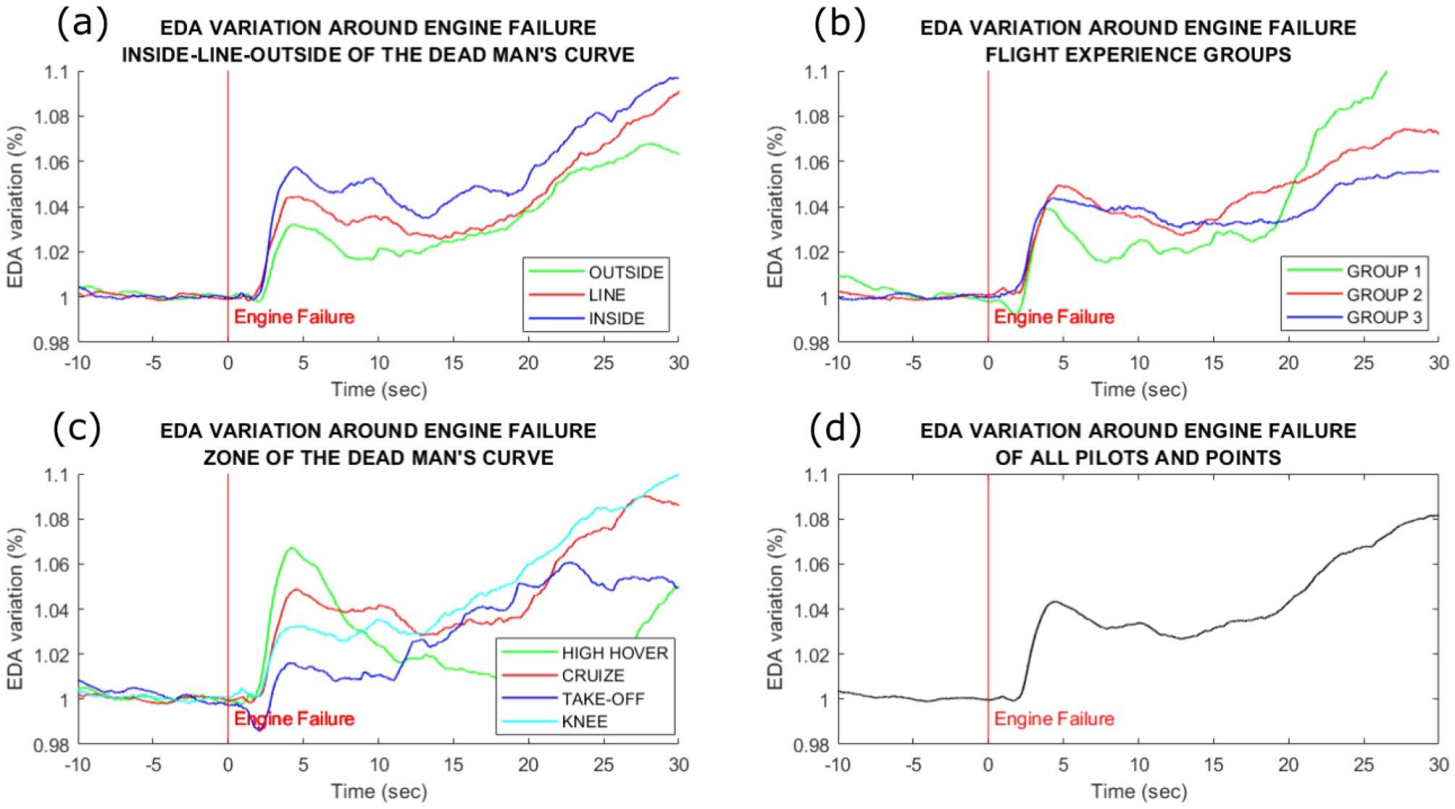
Electrodermal activity modulation after the engine failure: **(a)** mean EDA of maneuvers inside, outside or at the line limit of the height-speed diagram. **(b)** Mean EDA between different groups of pilot expertise: Group 1 (test Pilots with extensive autorotation experience), Group 2 (test Pilots with lesser autorotation experience) and Group 3 (operational pilots with lesser autorotation experience). **(c)** Mean EDA results for the four zone regions. The high-hover amplitude was significantly higher than Take-off and Knee amplitudes. **(d)** Mean EDA response around the engine failure for all maneuvers. We emphasize that these averaged figures are used only to facilitate an overview of the data. For statistics purposes, the effects of repetitions were incorporated in a linear mixed effect model.

The EDA response reached the established criteria in 170 maneuvers (89%). The average EDA responses are shown in Fig. 6d for better visualization of the process, although we utilized amplitude data for statistics tests. When those maneuvers were averaged, we were able to perceive signal modulation consisting of an augmented signal recorded around 2 s after the engine’s failure. This was a time point that coincided with instants of increased workload. There was a second rise, though of a non-canonical shape, after around 20 s.

Within the classical separation of the maneuvers, there was a statistically significant difference between the amplitude of EDA responses in the Inside versus the Outside area of the curve (Est = 0.294; *p* = 0.0395), Fig. 6a. Considering the zone separation, there were also significant contrasts (High-hover > Knee: EST = 0.373, p = 0.0291) and (High-hover > Take off = 0.537, p = 0.0291), Fig. 6c. When comparing groups of pilots of diverse previous experience, there was no significant difference in the EDA amplitude, Fig. 6b.

**Figure 7:**
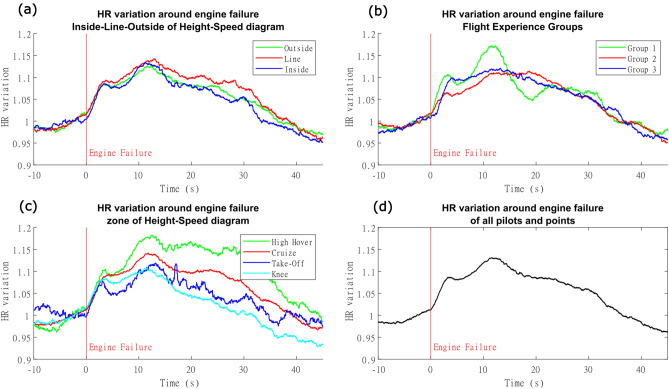
Heart rate changes around the engine failure: **(a)** HR changes during maneuvers inside, outside or at the line limit of the height-speed diagram. **(b)** HR changes between different groups of pilot expertise: Group 1 (test pilots with extensive autorotation experience), Group 2 (test pilots with lesser autorotation experience) and Group 3 (operational pilots with lesser autorotation experience). (c) HR changes for the four zone regions. The high-hover had the highest area under the curve, being followed by cruise. Take-off and Knee were not significantly different. **(d)** Average percent change of HR around the engine failure for all maneuvers. We emphasize that these averaged figures are used only to facilitate an overview of the data. For statistics purposes, the effects of repetitions were incorporated in a linear mixed effect model.

The average percent variation of heart rate (HR) shown in Fig. 7d indicates the dynamic changes over time that led us to use as a numeric parameter the area under the curve (AUC) representing the percent change of heart rates. No significant differences in heart rate AUC curves appeared when autorotation phases were compared with classical separation, Fig. 7a. There were significant differences when the proposed grouping phases were compared (Fig. 7c), namely [(High-hover > Cruise: EST = 1.3272, *p* = 0.0384); (High-hover > Knee = 3.2784, *p* < 0.0001); (High-hover > Take: EST = 3.3462, *p* = 0.0001); (Cruise > Knee = 1.9517, *p* = 0.0001); (Cruise > Take-off: EST = 2.019, *p* = 0.0077)]. The exception was Knee versus Take-off (Knee > Take-off: EST = 10.8; *p* = 0.1644). There were also no significant differences among pilot groups of diverse previous experience, Fig. 7b.

Respiratory Frequency data show an overall decrease in RF after the engine failure. However, there was no clear pattern for different flights carried out by the same pilots. For this reason, we were unable to identify and explore characteristic features and perform statistical analyses. The recorded data of the participants’ flights are available as supplementary material.

Overall, CPF and EDA had different modulations for both the classical partition of the Height-Speed diagram (O-L-I) and for the proposed one (Take-off-High-hover-Knee-Cruise). The HR had different responses only in the proposed partition. None of the measures were capable of show statistical differences in the group’s responses.

## Discussion

After a helicopter engine failure, the pilot performing an autorotation maneuver has to perceive the failure, interpret the alarms, control the aircraft angular accelerations, completely lower the collective command, adjust the speed and ultimately find a place to land^45^. All these processes must occur within approximately one second^29^, and, therefore, the maneuver is associated with great stress and increased workload.

One of the main functions of the test pilots is to report his or her opinion of the handling qualities of the aircraft after a flight^31^. The qualitative opinions of the test pilots are based on the same methodology proposed by the ADS-33^24^, where the MTE are evaluated through the HQR scale. This scale considers, among several factors, the use of rating scales of workload, such as the Cooper-Harper Scale^17^. In addition, vibration assessment data, turbulence, PIO, field of view and control displacements (frequency and amplitudes) are evaluated to better define the HQR of the task being performed. These data have already confirmed the influence of the workload in defining the limits of the Height-Speed diagram and are currently used during test flights to establish its limits^31,46^.

Another subjective assessment scale, the NASA-TLX^20^, has already been used in conjunction with physiological sensors^47^. Although this scale is widely accepted in the academic environment, it does not optimally evaluate autorotation, because its evaluation time and completion take much longer than the execution time of the maneuver itself and its evaluation criteria are also excessively subjective.

The main goal of this study was to complement the subjective workload assessment during autorotation by introducing objective measurable parameters. These candidate parameters were success rate, reaction time, CPF, EDA, heart rate, and respiration. These parameters can be correlated to the flight mechanics as well as to the pilots’ physiological responses.

We believe that the acquisition of many physiological signals synchronized to the Flight Test Instrumentation in a real flight provides novel knowledge to the aviation area. Even though the scenario is particularly challenging given that most physiological data are robust and consistent with the expected modulation after a sudden engine failure. Both the EDA and HR have showed abrupt increases after the engine failure, indicating that they could become physiological markers of the pilot’s workload. As for the respiration frequency, we have noticed instantaneous reduction, as opposed to the RF increase commonly reported in situations of workload ^48–51^. In addition, we could not notice a pattern when two different flights of the same pilot were analyzed. This may be due to some contamination as speaking took place shortly after the autorotation maneuvers. Furthermore, there is an intrinsic difference between the time scale of the signal and the event, i.e., the relatively low frequency of respiration can be difficult to assess during very fast events such as the autorotation maneuver after an engine failure. Therefore, no further analyses were performed for this signal.

Other studies have already measured workload by means of EDA tonic level for driving^52^ and air-traffic controlling^53^. However, we opted against the use of this parameter because of the short time of the landing maneuver after engine failure, as the signal lags to return to baseline levels. Instead, we analyzed the peak amplitudes preceding the event, given that it was robustly recorded in 90% of the maneuvers. One limitation of this analysis is the manual inspection for delimiting the instants of EDA’s derivative change, response start, and peak. Manual analyses are always prone to biases, however, it is still unknown how this procedure could be automatized^38^. In addition to that, there are reports of an increased EDA amplitude when the stimulus is related to a motor action^54^, which could potentially contaminate our recordings. However, since all maneuvers involved a similar action from the pilots, we expected that such change would occur in homogeneous ways in the recorded signals. Finally, our results did not seem to indicate a ceiling effect in EDA amplitude, as opposed to results from a flight simulation in a multitasking operation^52^.

One striking result from the HR percent change is the similarity between average responses when the two different flights of the same pilot are compared, which is suggestive of a signature response for each individual (supplementary files), although differences between pilots were seem. Our results indicated a robust modulation of the HR percent change following the engine failure (Fig. 7). Traditionally, in similar contexts (real and simulated flights), only segments with high workload such as take-off and landing are distinguishable from other flight periods ^55–57^, which suggest that a great amount of workload difference would be necessary to translate in HR, constituting a limitation for this marker as indicator of moderate workloads.

For the Success Rate assessment, international processes and standards such as FAR-27^12^, CS-27^58^ and RBAC-27^59^ establish that a single-engine helicopter must allow safe landing in autorotation, without requiring exceptional piloting skills. In our campaign, the test pilots from Group 2 had a success rate below those with the experienced pilots from Group 1, but the rate was above than operational ones from group 3 (Table 3), indicating that, despite the flight experience, the familiarity in performing the autorotation maneuvers appears to be more relevant.

Considering that: (1) all pilots involved in this flight test campaign were fully qualified and trained; (2) the aircraft used in the tests is certified under the international standards; and (3) all procedures provided by the aircraft’s operating manuals were fully executed; the results of the Success Rate, especially from Group 2 and 3, reveal that a significant part of the autorotations could not be completed safely. It indicates that there may be some gaps in the referred certification standards, particularly in the points where subjective evaluation is preponderant. The objective workload measures as those reported here may help in reducing the subjectivity of the ratings currently used to assess the workload and to better frame the effective safety of the different points of the Height-Speed Diagram.

Nevertheless, we recall that none of the objective workload parameters evaluated in this study (CPF, EDA, HR percent change) were capable to evidence the different levels of pilot experience, such as can be achieved by subjective questionnaires. In addition to that, according to the certification standard for this helicopter category (FAR-27), the reaction time must be either one second or the nominal reaction time for pilots, whichever is greater^12^. Overall, in the present helicopter flight test campaign, only two of the pilots had average reaction times compatible with the standard (Table 3), suggesting that the current certification processes may need an upgrade and the process of certification may need to be revised.

As for the classical partition of the Height-Speed Diagram (Outside, in Line, and Inside), our results indicated a statistically significant difference between the perceived workload, such that all areas can be characterized as distinct from one to another. In this scenario, when we evaluated the candidate objective markers, neither CPF nor the heart rate indicated similar modulation to the subjective workload assessment. However, for the EDA, the LME indicated trends distinguishing the outside areas of the curve from the inside and in line partitions.

Our findings corroborates with proposals supported by our group^30^ and by others^10^ that the workload is a significant parameter in the construction of the Height-Speed diagram. Our results also indicate that a pure mathematical model is not enough to predict the unsafe areas of this diagram, since it does not consider the workload at performing the autorotation maneuvers, nor the efficiency of the main rotor in converting the gravitational and kinetic energy into the main rotor RPM speed^13^. This result can be mainly attributed to the wide variations of attitude in the high-hover maneuvers and during the failures at low height and low speed (Knee and Take-off), when part of that energy accumulated is lost due to huge attitude variations. The greater the variation in the aircraft’s attitude, especially longitudinal (*Θ*), the higher is the loss of Nr and the greater the difficulty of recovering the main rotor RPM, that influences the performance of the helicopter's rotor^27^. The physical behavior of Nr recovery is directly related to the torque (Q) applied at the time of engine failure, which is an operational component, and to the moment of inertia of the rotor (I_R_)^60^, which is an intrinsic component of helicopter engineering design and manufacturing.

Following the flight tests’ evaluation doctrines, the workload quantification is primarily, and directly, related to the use of flight controls during the task^46^. This is confirmed by our results, as a peak in CPF emerged just after engine failure (Fig. 5—Cumulative Power Frequency of helicopter pilots carrying out in flight autorotation at Height-Speed diagram. (a) Pilots’ mean CPF at each phase of the classical partition of the Height-Speed diagram (Inside, Outside or at the Line limit). (b) Mean cumulative CPF of different groups of pilots according to their previous experience: Group 1 (test Pilots with extensive autorotation experience), Group 2 (test Pilots with lesser autorotation experience) and Group 3 (operational pilots with lesser autorotation experience). (c) Mean CPF of all pilots at the four proposed zones. Notice a difference between High-hover and Cruise and a similarity between Take-off and Knee zone. (d) sum of the global CPFs of all flights, indicating peak CPF responses at the time of engine failure.), that led us to hypothesize another segmentation of the Height-Speed diagram into four partitions (High-hover, Cruise, Knee and Take-off). In the High-hover Zone, the pilot must perform larger displacements of flight controls, especially on the longitudinal cyclic control, to recover the main rotor RPM and reach the recommended airspeed for performing the flare maneuver. In this partition, the flight maneuver profiles are distinct in comparison with other regions, since a 25° pitch-down maneuver is required as soon as the engine has failed. This task requires a high degree of concentration, frequent and extensive use of flight controls, and close attention to the NR overspeed control.

In the Knee, the aircraft has low energy, both in speed and in height (kinetic translational and gravitational potential energies, respectively). Besides, the pilot has few seconds to react on flight controls, since the helicopter presents a high rate of descent in these conditions, at an already low height flight profile, which imposes an important time constraint. As for the Cruise there are less restrictions on height and speed parameters than previous partitions, which accommodates a longer pilot’s reaction time and requires lower amplitudes and frequencies in the use of controls. Finally, for the Take-off there is a lower demand for flight controls usage to land. In fact, in this partition, the aircraft has little kinetic translational energy to be converted into the rotational kinetic energy to keep the main rotor RPM, which leads the pilot to level the aircraft, not being able to perform speed reduction maneuvers^31^.

The subjective workload assessment indicated a significant difference only between Knee and Cruise, and a trend in Take-off. Nevertheless, the CPF was significant at distinguishing the regions from one another, and the HR percent change was a parameter that was almost as successful, failing only to separate Knee and Take Off partitions. However, the EDA was capable to distinguish these partitions from one another, as well as each of them from High-hover. Our results point to a more nuanced description of the workload around the Height-Speed diagram, in comparison to the traditional approach. Nevertheless, we emphasize that especially the physiological markers should be used with caution when distinguishing different flight phases. Other studies reported no differences in EDA for distinct flight segments other than take-off, landing and touch and go^55^. In addition, significant differences in HR were reported only during the more demanding segments of the flight^55–57^.

Even though there seems to be a visual difference regarding the pilot’s experience with CPF peak (Fig. 5b), EDA (Fig. 6b), and Heart Rate (Fig. 7b), there was no significant increase in the statistical models using that variable. Further work with a higher number of repetitions and/or participants should be conducted to investigate this findings.

Therefore, our objective of assessing and differentiating the workload levels to which pilots are subjected in autorotation maneuvers was partially successful, we managed to describe the most robust and sensitive physiological sensors for this purpose.

Our results suggest that all objective measures evaluated in this work can be estimated with continuous signals recorded during flight. Continuous recording offer an opportunity to evaluate the aircraft since their development phase by providing more refined user requirements especially those relative to flight experience in emergency scenarios. For example, enhanced cabin cockpits that present the most required information for each flight phase in a personalized way—a type of interactive panels, or autopilots that focus their capabilities on reducing the highest workload determined by employing the methods proposed by this study.

The combination of objective measurement parameters, such as the analyses of the flight controls displacements, and the pilot’s physiological responses indicate great potential for complementing the subjective evaluations currently employed. This can also improve processes, indicating optimization and improvement paths more objectively and efficiently, contributing to the uplift and increase of flight safety.

The physiological results can assist the creation of methods of monitoring the pilots' attention status during real-time flight, allows a complimentary assessment of fatigue, attention level, and the ability to perform tasks in stressful situation requiring high demand of workload. Beyond that, such methods can allow objective monitoring of the efficiency of pilot's psychomotor training through the technique of analysis of the movements of the flight controls, contributing to the pilot's selection processes, and in operational status progression when the pilot could become an instructor or commander of the aircraft.

The present methodology can contribute to make the training of pilots more agile and efficient besides providing a new area of study: the assessment of the psychomotor and emotional conditions of the pilots in real-time. It could help in the development of control tools for fatigue, loss of attention, and the strange behaviors that can affect flight safety.

An important limitation of this study is related to the small number of participants and the relatively low number of trials performed by each of them, when compared to typical values involved in common physiology studies. This limitation stems from the high level of training required for pilots to participate in this type of flight campaign as well as the costs associated with each flight hour.

To future work, we believe that these sensors and parameters can be analyzed together, since our acquisition has been synchronized, allowing the extraction of more subtle and previously unobservable patterns as those provided by individual sensors. Although we have made some unsuccessful attempts in this direction, we believe that multimodal analysis can enrich the description of the phenomenon, especially if physiological and aircraft data can be combined.

## Supplementary Information

Supplementary Information.

## Data Availability

The datasets generated during and/or analyzed during the current study are available from the corresponding author on reasonable request.
