# Supplementary material for: Quantitative assessment of pilot-endured workloads during helicopter flying emergencies: an analysis of physiological parameters during an autorotation
Source: Sci Rep. 2021 Sep 6;11:17734. doi: 10.1038/s41598-021-96773-y (PMC8421440; doi:10.1038/s41598-021-96773-y)

Supplemental material of “**Quantitative assessment of pilot-endured workloads during helicopter flying emergencies: an analysis of physiological parameters during an autorotation”**

Jose´ Ricardo Silva Scarpari1,2,+, Mauricio Watanabe Ribeiro3,+, Camila Sardeto Deolindo3, Maria Adelia Albano Aratanha3, Donizeti de Andrade1, Carlos Henrique Quartucci Forster1, Jose´ Ma´rcio Pereira Figueira2, Fernando Lucas Soares Corrêa^2^, Shirley Silva Lacerda3, Birajara Soares Machado3, Edson Amaro Ju´nior3, Joa˜o Ricardo Sato3,4, Elisa Harumi Kozasa3,*◦*,*, and Roberto Gil Annes da Silva1,*◦*

*[ehkozasa@gmail.com](about:blank)

1Instituto Tecnolo´gico da Aerona´utica, Sa˜o Jose´ dos Campos, 12228-900, Brazil

2Instituto de Pesquisas e Ensaio em Voos (IPEV), Sa˜o Jose´ dos Campos, 12228-900, Brazil

3Hospital Israelita Albert Einstein, Brain Institute, Sa˜o Paulo, 01425-001, Brazil

4Universidade Federal do ABC, Sa˜o Bernardo do Campo, Brazil

+ Indicates co-first authorship; º Indicates co-senior authorship

| Supplemental Material #1: Results of Linear Mixed Models  Linear Mixed Models Constrasts | | | | | | | | | | | |
| --- | --- | --- | --- | --- | --- | --- | --- | --- | --- | --- | --- |
|  | HQR | |  | logEDA | |  | AUC HR | |  | CPF | |
| Classical Regions | estimate | p-value |  | estimate | p-value |  | estimate | p-value |  | estimate | p-value |
| Line - Outside | 0.5 | *0.0246* |  | 0.157 | *0.2402* |  | 0.74 | *0.4456* |  | 15.7 | *0.0487* |
| Inside - Line | 1.6 | *<.0001* |  | 0.137 | *0.2795* |  | -0.58 | *0.4678* |  | 5.5 | *0.5019* |
| Inside - Outside | 2.1 | *<.0001* |  | 0.294 | *0.0395* |  | 0.16 | *0.761* |  | 21.2 | *0.0157* |
|  |  |  |  |  |  |  |  |  |  |  |  |
| Proposed Zones | estimate | p-value |  | estimate | p-value |  | estimate | p-value |  | estimate | p-value |
| Cruise - High Hover | -0.493 | *0.2222* |  | -0.2 | *0.1806* |  | -1.32 | *0.0403* |  | -63.8 | *<.0001* |
| Cruise - Knee | -0.786 | *0.0174* |  | 0.172 | *0.1806* |  | 1.96 | *0.0001* |  | 25.3 | *<.0001* |
| Cruise - Take-off | 0.148 | *0.6994* |  | 0.337 | *0.1149* |  | 2.03 | *0.0074* |  | 36.2 | *<.0001* |
| High Hover - Knee | -0.293 | *0.4706* |  | 0.373 | *0.0293* |  | 3.27 | *<.0001* |  | 89.1 | *<.0001* |
| High Hover - Take-off | 0.642 | *0.2222* |  | 0.537 | *0.0293* |  | 3.35 | *0.0001* |  | 100.0 | *<.0001* |
| Knee - Take-off | 0.934 | *0.0565* |  | 0.165 | *0.3578* |  | 0.07 | *0.9202* |  | 10.8 | *0.1633* |
|  |  |  |  |  |  |  |  |  |  |  |  |
| Experience | estimate | p-value |  | estimate | p-value |  | estimate | p-value |  | estimate | p-value |
| Group1 - Group2 | -0.532 | *0.0974* |  | -0.221 | *0.5478* |  | 0.76 | *0.9377* |  | -7.8 | *0.7236* |
| Group1 - Group3 | -1.501 | *0.0029* |  | 0.272 | *0.5478* |  | 0.12 | *0.9377* |  | 4.2 | *0.7236* |
| Group2 - Group3 | -0.969 | *0.0095* |  | 0.493 | *0.5101* |  | -0.65 | *0.9377* |  | 12.0 | *0.7236* |

Supplemental Material #2: Mean Percent Change of Electrodermal Activity


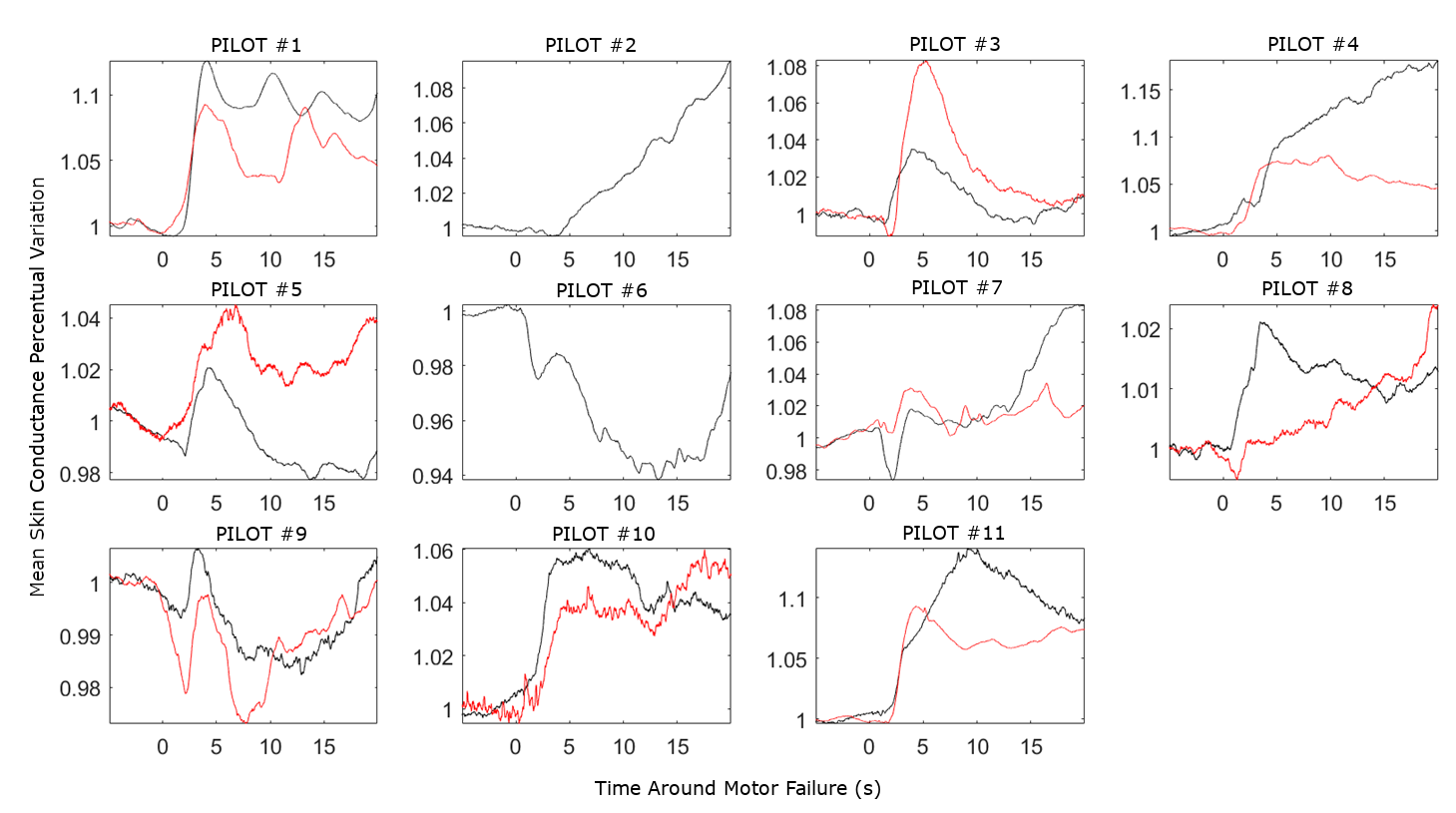


Supplemental Material #3: Mean Percent Change of Heart Rate


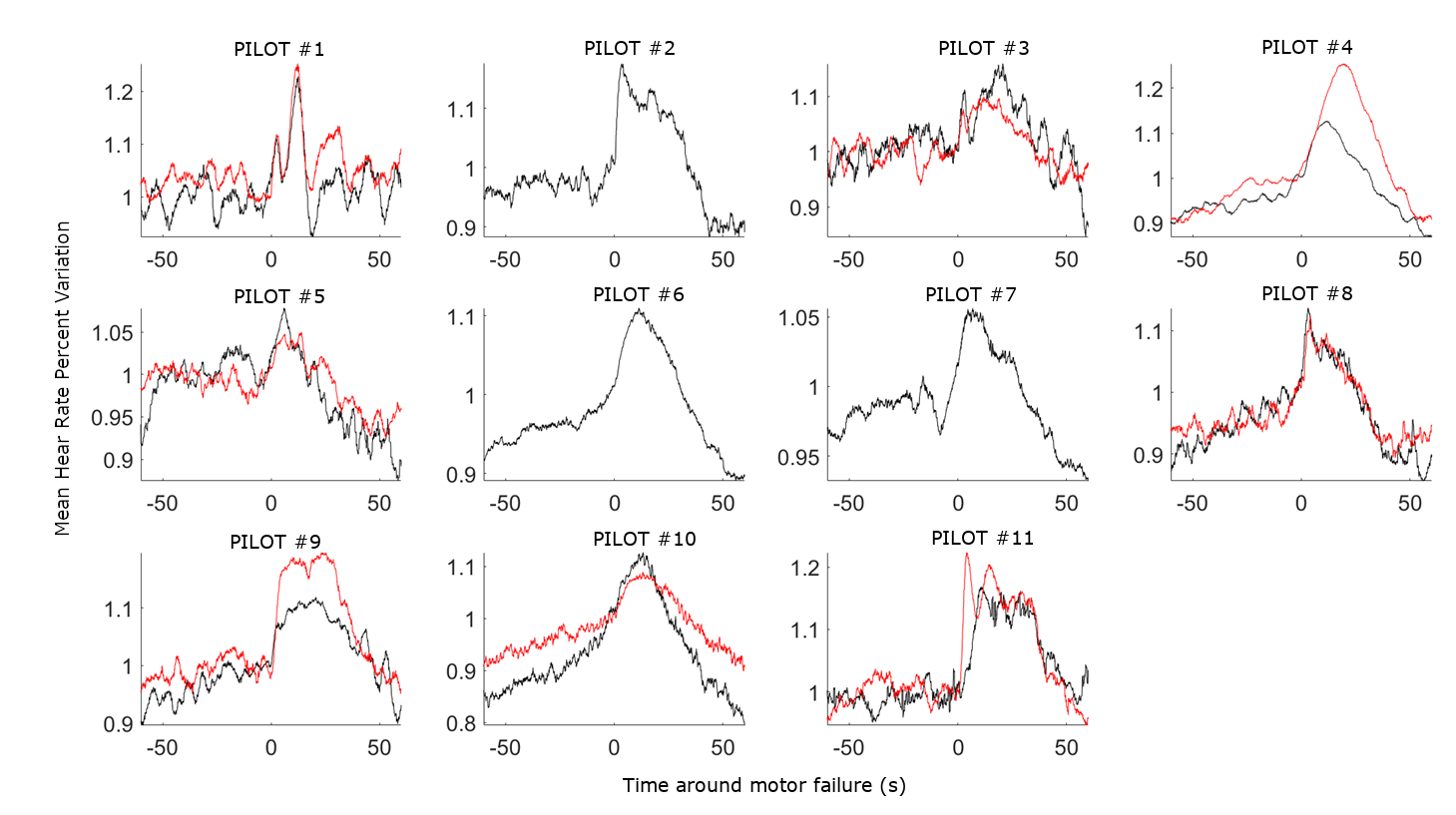


Supplemental Material #4: Mean Promediated Frequency


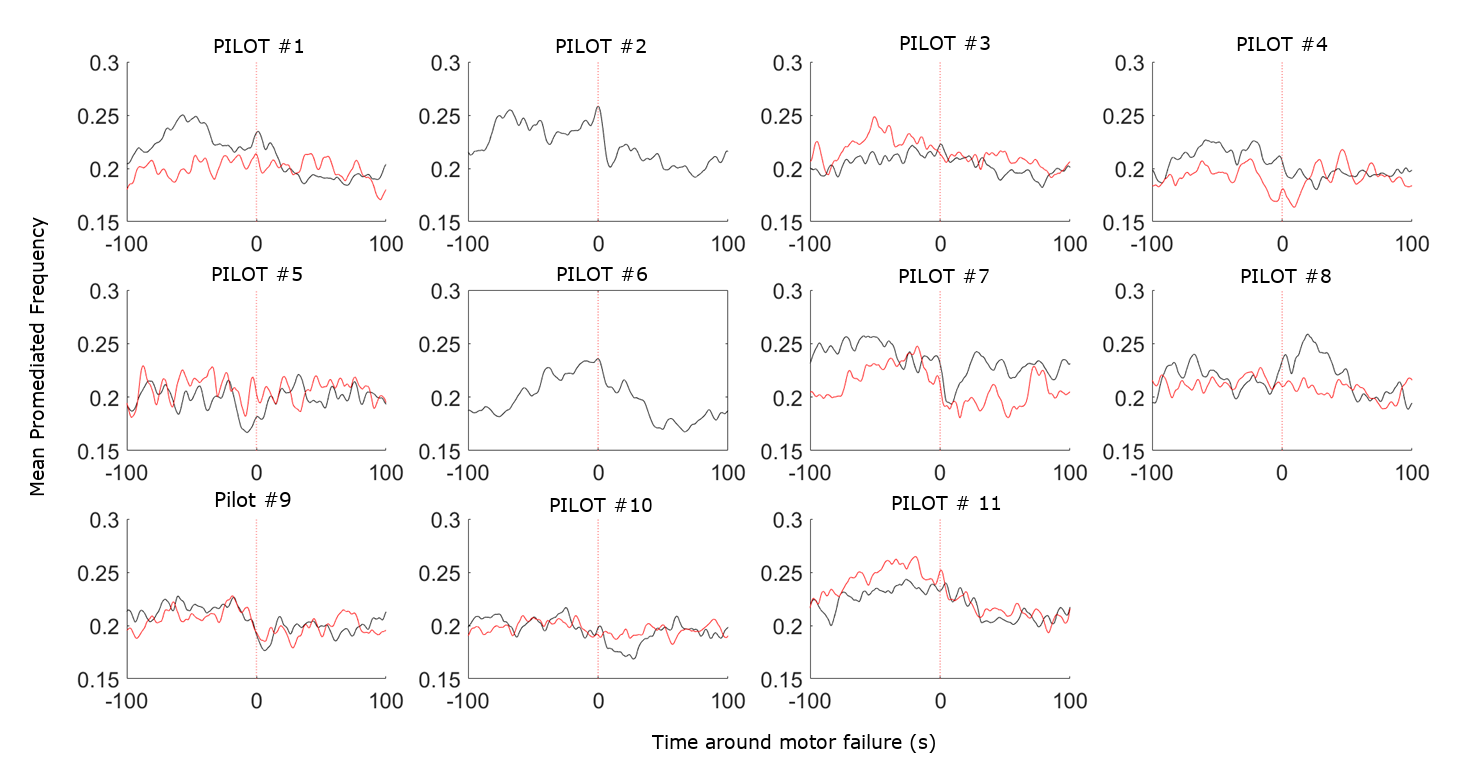

Supplement: Supplementary file 1 — Supplementary Information. [file 41598_2021_96773_MOESM1_ESM.docx]
